# Unusual cause of acute sciatica

**DOI:** 10.11604/pamj.2014.18.85.4185

**Published:** 2014-05-26

**Authors:** Brahim Eljebbouri, Ali Akhaddar

**Affiliations:** 1 Department of Neurosurgery, Mohammed V Military Teaching Hospital, University of King Mohammed V Souissi, Maroc

**Keywords:** Acute sciatica, CT-scan, B acute lymphoblastic leukemia

## Image in medicine

This 32-year-old-man was admitted to the emergency department because of S1 left acute sciatica. An emergency CT-scan showed a highly probable appearance of herniated disc in L5-S1 level (A). The patient was operated without finding disc herniation, but an aspect of anterior and posterior epidural abscess compressing the dural sheath at L5-S1. Medullary MRI showed after the up and the down extention of disease process (B). Histological examination of samples found a B lymphoblastic leukemia (C). The patient died 3 weeks after because of systemic infection. After review of literature, it was never been described that the sciatic pain can be a mode of revelation of B acute lymphoblastic leukemia. Only two cases reported this mode of revelation but for lymphoma requiring a surgical intervention -as our case- firstly to relieve the patient by a radicular decompression and secondly for histological diagnosis.

**Figure 1:**
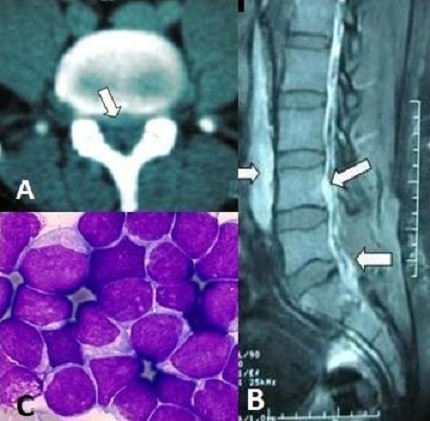
(A) CT-scan showed highly probable appearance of herniated disc in L5-S1 level; (B) Medullary MRI showed after the up and the down extention of disease process; (C) Histological examination of samples found a B lymphoblastic leukemia

